# A Series of Patients with Genodermatoses in a Reference Service for Rare Diseases: Results from the Brazilian Rare Genomes Project

**DOI:** 10.3390/genes16050522

**Published:** 2025-04-29

**Authors:** Carlos Eduardo Steiner, Maria Beatriz Puzzi, Antonia Paula Marques-de-Faria, Ruy Pires de Oliveira Sobrinho, Vera Lúcia Gil-da-Silva-Lopes, Carolina Araújo Moreno, The Rare Genomes Project Consortium

**Affiliations:** 1 Departamento de Genética Médica e Medicina Genômica, Faculdade de Ciências Médicas, Universidade Estadual de Campinas (Unicamp), Campinas 13083-888, SP, Brazil; apmfaria@unicamp.br (A.P.M.-d.-F.); ruypires@unicamp.br (R.P.d.O.S.); vgslopes@unicamp.br (V.L.G.-d.-S.-L.); carolinaaraujomoreno@yahoo.com.br (C.A.M.); 2 Disciplina de Dermatologia, Departamento de Clínica Médica, Faculdade de Ciências Médicas, Universidade Estadual de Campinas (Unicamp), Campinas 13083-888, SP, Brazil; beatrizpuzzi@gmail.com; 3 Serviço de Genética Molecular, Departamento de Medicina Laboratorial, Hospital Israelita Albert Einstein (HIAE), São Paulo 05652-900, SP, Brazil

**Keywords:** rare diseases, genodermatoses, ectodermal dysplasia, ichthyosis, albinism, tuberous sclerosis complex, incontinentia pigmenti, genome sequencing

## Abstract

Background/Objectives: Genodermatoses are genetic conditions with clinical symptoms manifesting in the skin and adjoining tissues, individually rare but comprising a large and heterogeneous group of disorders that represents 15% of genetic diseases. This article discusses the results of individuals with genodermatoses from a reference center for rare diseases studied through whole genome sequencing conducted by the Brazilian Rare Genomes Project between 2021 and 2023. Methods: A retrospective case series with data comprising sex, age at first assessment in the hospital, family history, clinical findings, and molecular results. Results: Excluding neurofibromatosis type 1, Ehlers–Danlos syndrome and RASopathies are discussed elsewhere. Diagnoses in this work comprised ectodermal dysplasias (*n* = 6), ichthyosis (*n* = 4), albinism (*n* = 4), tuberous sclerosis complex (*n* = 4), and incontinentia pigmenti (*n* = 3), in addition to 11 others with individual rare conditions. The sex ratio was 17:16 (M:F), consanguinity was present in 6/33 (18%), and the age at the first evaluation ranged from neonatal to 26 years (median 13.65 years). Negative results were 3/33 (9%), novel variants were 17/41 (41.4%), and 7/30 (23%) presented initially with a double molecular diagnosis, three confirming composed phenotypes. Conclusions: Besides reporting 17 novel variants in 14 genes (*BLM*, *CACNA1B*, *EDA*, *ELN*, *ENG*, *ERC6*, *EVC2*, *PNPLA1*, *PITCH1*, *PORCN*, *SIN3A*, *TP63*, *TYR*, and *WNT10B*), the study also identified three atypical clinical presentations due to dual diagnoses, and the c.454C>T variant in the *SDR9C7* gene, previously reported only in dogs, was, for the first time, confirmed as causative for ichthyosis in humans.

## 1. Introduction

The Clinical Genetics Service at the University of Campinas (CGS/Unicamp) was established in 1969 as the first in Brazil. Historically originating from a single outpatient clinic, the service has progressively evolved into specialized services over the 50 years of its activity as the number of patients and professionals increased following advances in medical genetics. Over time, it was organized into subspecialty areas, each one providing diagnostic evaluation, follow-up, and therapeutic management, when available, as well as genetic counseling. These subspecialty areas included dysmorphology, inborn errors of metabolism, hemoglobinopathies, oncogenetics, neurogenetics, disorders of sex development, neurodevelopmental disorders, skeletal dysplasias, and genodermatoses.

In April 2004, a genodermatoses outpatient clinic was established at the CGS/Unicamp, with the participation of two dermatologists, two clinical geneticists, and one pediatric neurologist, to provide specialized care for this patient group on a half-day basis, once a week. It remained active until December 2021, with over 450 patients being evaluated. Its activities ceased due to staff reductions and internal administrative restructuring. The patients in clinical follow-up were transferred to other outpatient clinics within the CGS/Unicamp.

Genodermatoses refers to genetic diseases, generally monogenic, that manifest clinically in the skin and adnexal tissues. Like most inherited disorders, they are individually rare but comprise a large and heterogeneous group representing 15% of genetic diseases worldwide. Genodermatosis lesions can be very visible, have a psychological impact on patients, result in social stigma, and negatively affect patients’ quality of life. Many genodermatoses lead to chronic disability, others to death [1].

The Brazilian Rare Genomes Project (BRGP) is a public-private initiative that incorporates whole genome sequencing (WGS) to improve the diagnosis of rare genetic diseases in Brazil, aiming to integrate genomic precision medicine into the Brazilian public healthcare system known as the Unified Health System (Sistema Único de Saúde; SUS) [2]. One of the goals is to reduce the lengthy process of diagnosing patients with rare disorders, as the average duration of the diagnostic odyssey in Brazil is 5.4 years (±7.9 years) [3].

The BRGP conducts molecular testing in two central laboratories for patients with rare diseases or hereditary cancer undergoing clinical investigation and/or follow-up at 21 of the 32 Reference Centers for Rare Diseases distributed nationwide [4]. During the first triennium of the project (2021–2023), over 8000 probands were recruited. In 2024, the Brazilian Ministry of Health provided additional funding to include new centers and 2000 individuals in an extension phase of the project.

In 2019, a Reference Center for Rare Diseases at the University of Campinas was officially accredited, following the Brazilian Policy for Comprehensive Care for Persons with Rare Diseases [5,6], incorporating the activities of the CGS/Unicamp and being one of the participating centers of the BRGP. Located in the Campinas metropolitan area, in the Southeastern and most populous region of the country, it is based in a teaching hospital, serving as the leading public tertiary healthcare service for a population of around 6 million.

This study aims to contribute to the understanding of rare diseases in Brazil by providing clinical and molecular data, ultimately leading to a better understanding of the epidemiology and genotypic and phenotypic variations of genetic disorders in this population. Different groups of conditions are being separately analyzed following the nosology of the BRGP, and for the present paper, data regarding dermatological and neurocutaneous disorders are presented.

## 2. Materials and Methods

This article analyzes clinical and molecular data from a case series of individuals with genodermatoses followed at the CGS/Unicamp between 2021 and 2023. Individuals were invited to participate in the BRGP research protocol after the Institutional Ethics Committee had approved it. Written consent was obtained from patients or their legal guardians before the procedures. Data were obtained from medical records and confirmed by patients and/or relatives; they included sex, age at first hospital assessment, family history, description of clinical and laboratory findings, and molecular results.

Molecular studies included WGS performed on DNA extracted from peripheral blood using an Illumina platform following mechanical fragmentation and a PCR-free protocol. Data were processed to detect point mutations, copy number variations (CNVs), and structural variants according to best practices for bioinformatics pipelines [2]. Quality metrics required a minimum coverage of 20× and at least 90% depth above 15×. The reference genome was GRCh38/hg38.

Variants were classified according to the American College of Medical Genetics criteria recommendations to standardize variant classification by 2023 [7,8], using the refinements proposed by the Sequence Variant Interpretation Working Group. Patients were included in this study if the variants were classified as pathogenic and likely pathogenic, or of uncertain significance (VUS) with a positive phenotypic correlation.

The analysis and discussion utilized the Franklin by Genoox community platform (https://franklin.genoox.com, accessed on 23 January 2025), PubMed (https://pubmed.ncbi.nlm.nih.gov/, accessed on 23 January 2025), and ClinVar (https://www.ncbi.nlm.nih.gov/clinvar/, accessed on 23 January 2025) databases. A variant was considered novel if it had not been previously published or registered in the ClinVar database [9]. When a variant was present in ClinVar in only one assertion, which corresponded to the patients reported in this study, it was still considered a novel variant, since the results of the BRGP were also deposited in the ClinVar database.

Incidental or secondary findings [10] were not discussed in the present study.

## 3. Results

Of the 376 probands recruited by our group for the BRGP from 2021 to 2023, 79 (21%) were included in the project due to primary cutaneous or neurocutaneous manifestations, including neurodevelopmental disorders (NDDs).

Among these 79 individuals, the largest group of patients with genodermatoses was neurofibromatosis type 1, corresponding to 38% of all individuals who underwent WGS in the cohort. These results were previously discussed and are being submitted for publication separately.

The second largest diagnostic group was Ehlers–Danlos syndrome, comprising three individuals with classic or type 1, one with vascular, one with kyphoscoliotic, two awaiting final analysis, and three with hypermobility type (all three with negative results), which will be presented in the future along with other connective tissue disorders diagnosed in this center.

The study also identified six individuals with disorders comprising genes that encode components or regulators of the Ras/mitogen-activated protein kinase (MAPK) pathway (RASopathies), including two with Noonan syndrome, two with Noonan syndrome with multiple lentigines, one with Noonan syndrome-like, and another with neurofibromatosis-Noonan phenotype. These results were also previously discussed.

Thus, data on the remaining 33 patients are presented in this article.

The sex ratio was 17:16 (M:F). Consanguinity was present in 6/33 (18%) families, and the age at the first evaluation in the service ranged from 21 days to 26 years, with a median of 13.65 years.

Regarding the molecular results, 3/33 (9%) individuals had a negative result, 17/41 (41.4%) variants are novel, and 7/30 (23%) patients presented with a double molecular diagnosis. The 17 novel variants were identified in 14 genes, with two variants each in *CACNA1B*, *ERC6*, and *TP63*, and one variant each in *BLM*, *EDA*, *ELN*, *ENG*, *EVC2*, *PNPLA1*, *PORCN*, *PTCH1*, *SIN3A*, *TYR*, and *WNT10B*; two were copy number variations (CNV), and the remaining were single-nucleotide variants (SNVs).

The main groups of conditions among the 33 probands were ectodermal dysplasias (*n* = 6; Table 1), ichthyosis (*n* = 4; Table 2), albinism (*n* = 4; Table 3), tuberous sclerosis complex (*n* = 4; Table 4), and incontinentia pigmenti (*n* = 3; Table 5). Other rare conditions were individually diagnosed and are presented in Table 6 as a miscellaneous group.

## 4. Discussion

The sex ratio distribution in this series was nearly even, reflecting the fact that most disorders diagnosed in the cohort followed autosomal inheritance, except for one individual with an X-linked recessive disease (patient 1) and four with X-linked dominant conditions (patients 19, 20, 21, and 26).

Consanguinity was reported in six families, including the four probands presenting with ichthyosis (patients 7–10), one with classical albinism (patient 11), and one with Cowden syndrome (patient 27). For the five formers, the detected variants were in homozygosity, as expected in the offspring of consanguineous parents. For patient 27, who also exhibited the highest inbreeding coefficient, as he was born to a father–daughter incestuous relationship, it was considered casual and not related to the molecular diagnosis. Family 11 also exhibited a pseudodominant inheritance, as the proband, her dizygous twin brother, and the mother were all albinos; the mother was born to first-degree cousins and had also married a cousin of unspecified degree from the same small village.

Contrary to expectations, four patients (3, 5, 14, and 24) with rare homozygous variants, two novel (patients 5 and 24), were born to parents who denied consanguinity and were not from the same village or neighboring localities. In a particular situation, the variant in patient 14 was also associated with uniparental isodisomy, which thoroughly explains his homozygous status. For the remaining three individuals, the possibility of a more distant consanguinity in these cases was further investigated by determination of runs of homozygosity regarding number (NROH), sum (SROH), and fraction (FROH), which were low for individuals 3 (NROH = 4; SROH= 13.1 Mb; FROH = 0.5%) and 24 (NROH = 2; SROH = 8.5 Mb; FROH = 0.3%), but confirmed consanguinity for patient 5 (NROH = 38; SROH = 597 Mb; FROH = 20.8%).

Double molecular diagnoses are expected to occur in 2–7.5% of cases diagnosed through comprehensive genomic testing, with a higher frequency in consanguineous families. However, double diagnoses can also occur due to two pathogenic variants in autosomal dominant disease genes, usually arising as de novo events [29,30]. In the present study, no cases of double molecular diagnoses were identified in the consanguineous families; however, they were found in seven of the 30 positive WGS tests, resulting in a frequency of 23%, higher than the expected rate. In two patients (4 and 6), the second molecular finding was set aside after reversal phenotyping, and in patient 19 it could not be excluded, since both conditions can explain the dental abnormalities that she presents. As previously discussed, patient 14 exhibited homozygosity for a variant in the *OCA2* gene resulting from uniparental isodisomy. For the remaining three cases (patients 3, 5, and 13), an addictive effect of both conditions explained their clinical presentation. Even considering this analysis, the frequency of 3/30 (10%) remains above the expected rate for double diagnoses suggested in the literature, probably indicating that this value might be higher when a thorough genomic analysis is performed.

Regarding the ectodermal dysplasias group, patient 1 has typical features of hypohydrotic ectodermal dysplasia, with a negative family history for similar cases. Patient 2 was initially challenging to classify between Rapp–Hodgkin and Hay–Wells syndrome, but at least her clinical picture seems more compatible with the latter, as was patient 3, who also exhibited facial dysmorphism, tracheomalacia, and NDD, compatible with a double diagnosis of ectodermal dysplasia and DEGCAGS syndrome.

Individual 4 had the same variant as the patient described by Leduc et al. [12]. The clinical picture overlaps with the cardiologic and some dermatologic findings, but differs regarding neurologic findings, as their patient presented with microcephaly and NDD, whereas individual 4 had normal neurodevelopmental milestones, satisfactory school performance, and regular social interaction. Patient 4 died at the age of 13 years due to postoperative heart surgery complications.

Patient 5 presented with typical features of Ellis–van Creveld syndrome, including delayed neurologic milestones and learning disabilities, overlapping features with the second molecular diagnosis of chromosome 22q11.2 microduplication syndrome, a condition with highly variable phenotype, ranging from asymptomatic to severely affected individuals.

Besides the features of cranioectodermal dysplasia type 4, patient 6 had a pathogenic 235.7 Kb heterozygous deletion in 6q13-q13, encompassing exons 36 to 66 of the *COL12A1* gene. Variants in this gene are associated with autosomal dominant Ullrich congenital muscular dystrophy 2 and autosomal recessive Bethlem myopathy 2; however, the patient doesn’t exhibit clinical features of a muscle disorder at 13 years, and both conditions have a congenital onset. Therefore, reverse phenotyping was considered negative for this finding.

Concerning the second group of genodermatoses shown in Table 2, three individuals had isolated and one a syndromic form of ichthyosis, all born to consanguineous parents and presenting homozygous variants. Patients 7 and 8 manifested as congenital ichthyosiform erythroderma; the former had a previously described variant in the *NIPAL4* gene [16] and the latter a novel variant in the *PNPLA1* gene. The c.454C>T p. (Arg152Trp) variant in the *SDR9C7* gene, identified in patient 9 and classified as a VUS, was also previously reported in a homozygous state in Chihuahua dogs and suggested to be a candidate gene for ichthyosis in humans [17].

Sjögren–Larsson syndrome is a well-known form of syndromic ichthyosis. It is prevalent in the metropolitan region of Campinas, where the most common variant in the Brazilian population of the *ALDH3A2* gene (c.1108-1G>C) has been identified [31]. Patient 10 had a different variant, c.1443+1G>A, initially identified in 2017 under ClinVar SCV002500825 and classified as VUS. However, it was also later described in the Brazilian population in 2022 under ClinVar SCV002517549 and reclassified as a likely pathogenic variant.

Patients 11 and 12 had isolated classical albinism confirmed by variants identified in the *TYR* gene. On the other hand, patients 13 and 14 presented with albinism and NDD, and an initial diagnosis of oculocerebral hypopigmentation syndrome of Preus [32] was considered for both. Molecular results confirmed that both had a double diagnosis involving the *OCA2* gene, patient 13 with two additional variants in the *CACNA1B* gene responsible for an NDD with seizures and nonepileptic hyperkinetic movements, and patient 14 with Angelman syndrome due to a paternal isodisomy of chromosome 15 carrying a heterozygous 142.2 kb duplication comprising exons 3 to 19 of the *OCA2* gene. This association of albinism and Angelman syndrome was previously reported [23], including another case in the Brazilian population [33].

Four individuals were diagnosed with tuberous sclerosis: patient 15 with a SNV in the *TSC1* gene, patient 16 with a SNV in the *TSC2* gene, and patients 17 and 18 with deletions that also encompassed the *TSC2* gene. Patient 18 was previously submitted to molecular investigation through a gene panel sequencing that was negative, and the deletion involving exons 6 and 7 of the *TSC2* gene was only identified when he underwent WGS. All variants in this group are consistent with previous reports [34].

Patients 19–21 manifested typical features of incontinentia pigmenti, although only individual 21 is old enough to present with stage 4 of the disease, characterized by hypopigmented blaschkoid streaks. All three confirmed the deletion of exons 4 to 10 in the *IKBKG* gene through long-read sequencing, which is the most frequent genomic mechanism responsible for nearly 65% of the cases [27,35].

The following individuals, 22 to 33, formed a miscellaneous group involving several genodermatoses.

Epidermolysis bullosa is another relevant genodermatosis [36], which was underrepresented in this study, with only one diagnosed individual (patient 22) who was initially suspected of having incontinentia pigmenti. This underrepresentation can be explained by the fact that the Brazilian Association of Bullous Epidermolysis (DEBRA Brasil; https://debrabrasil.com.br/) facilitates access to molecular testing for patients and their families. Thus, several patients with epidermolysis bullosa seen in our service had a previous molecular diagnosis, and such cases were not included in the BRGP.

Patients 23 to 28 and 30 had a typical clinical presentation for each condition individually diagnosed, and no specific remarks on their cases are presented, except for the novel variants detected.

Patient 29 had a pathogenic heterozygous deletion of 4.35 Mb in 10q11.22q11.23, comprising 40 coding genes, 22 registered in the OMIM database. Together with a hemizygous variant in the *ERCC6* gene, the diagnosis of Cockayne syndrome type B was established. She had overall healthy conditions, but at the age of 26 years, she presented a sudden intense migraine episode evolving to hemiplegia, and during hospital admission, she developed total retrograde amnesia involving daily life activities and even the recognition of the close relatives. To the best of our knowledge, this has never been described in this condition.

Patient 31 exhibited clinical features suggestive of Rothmund-Thomson syndrome (RTS); however, the WGS test was negative. This is a genetically heterogeneous condition, with two primary known causative genes accounting for approximately 70% of cases: *ANAPC1* for RTS1 and *RECQL4* for RTS2. Recently, two other genes were identified: *CRIPT* for RTS3 [37] and *DNA2* for RTS4 [38]. Nonetheless, a considerable number of individuals remain negative for variants in those four genes, and patient 31 will be reanalyzed in the future.

Finally, patients 32 and 33 had manifestations of Klippel–Trénaunay syndrome and were included in the BRGP due to NDD manifestations. In both cases, the WGS results were negative, as is usually seen in this condition.

## 5. Conclusions

In this heterogeneous group of genodermatoses, the authors reinforced clinical and molecular findings in previously known conditions, in addition to reporting 17 novel variants in 14 genes (*BLM*, *CACNA1B*, *EDA*, *ELN*, *ENG*, *ERC6*, *EVC2*, *PNPLA1*, *PITCH1*, *PORCN*, *SIN3A*, *TP63*, *TYR*, and *WNT10B*). The study also identified three atypical clinical presentations due to dual diagnoses, and the c.454C>T variant in the *SDR9C7* gene, previously reported only in dogs, was, for the first time, confirmed as causative for ichthyosis in humans.

## Figures and Tables

**Table 1:** Individuals with ectodermal dysplasias diagnosed in the center, including sex, age at first evaluation, clinical findings, diagnosis, and gene variant(s).

# Sex Age	C	Clinical Synopsis Diagnosis, MIM	Gene (Transcript) Variant Zygosity, Classification	Reference
1 M 1 ^11^/_12_ y	-	Alopecia, hypohydrosis, hypodontia. Hypohydrotic ectodermal dysplasia, MIM: 305100	*EDA* (NM_001399.5) c.920dup p.(Glu308Argfs*9) Hemizygous, Likely Pathogenic	novel
2 F 1 ^4^/_12_ y	-	Cleft lip/palate, hypodontia, hypotrichosis, nail dysplasia, erythroderma, blepharophimosis, photophobia, bilateral SNHL. Rapp–Hodgkin syndrome, MIM: 603273	*TP63* (NM_003722.5) c.1715_1717del p.(Thr572del) Heterozygous, VUS	novel
3 M 2 ^5^/_12_ y	-	Short stature, microcephaly, facial dysmorphisms, decreased tear drainage, alopecia, fragile nails, generalized anhidrosis, cryptorchidism, multiple renal cysts, tracheomalacia, hearing impairment, severe NDD. Rapp–Hodgkin syndrome and DEGCAGS syndrome, MIM: 603273 and 619488	*TP63* (NM_003722.5) c.1991A>G p.(Asp664Gly) Heterozygous, VUS	novel
			*ZNF699* (NM_198535.3) c.1327C>T p.(Arg443Ter) Homozygous, Likely Pathogenic	[11]
4 M 3 ^4^/_12_ y	-	Partial atrioventricular septal defect, valvar dysplasia, prominent forehead, hypothricosis, oligodontia, conic incisor, hypothyroidism, nephrocalcinosis. Congenital heart defects and ectodermal dysplasia, MIM 617364	*PRKD1* (NM_002742.3) c.2134G>A p.(Val712Met) Heterozygous, VUS	[12]
			*SIN3A* (NM_001145358.2) c.2722C>T p.(Arg908Trp) Heterozygous, VUS	novel
5 M 6 ^1^/_12_ y	-	Short stature, rhizomelia, genu valgum, narrow chest, postaxial polydactyly, nail dysplasia, gingival defects, oligodontia, NDD. Chondroectodermal dysplasia (Ellis–van Creveld syndrome) and chromosome 22q11.2 microduplication syndrome, MIM 225500 and 608363	*EVC2* (NM_147127.5) c.50del p.(Gly17ValfsTer44) Homozygous, Pathogenic	novel
			seq[GRCh38] dup(22)(q11.21q11.21) NC_000022.11:g. (18948676_21110520)dup/3× Heterozygous, Pathogenic	[13,14]
6 F 2 ^10^/_12_ y	-	Short stature, short trunk, C1-C4 vertebral fusion, preauricular pit, hypertelorism, epicanthal folds, microretrognathia, early dental eruption, abnormal teeth, postaxial polydactyly, cubitus valgus, genu valgum, NDD. Cranioectodermal dysplasia type 4, MIM: 614378	*WDR19* (NM_025132.4) c.1250-1G>A p.? Heterozygous, Likely Pathogenic	ClinVar ID 802068
			*WDR19* (NM_025132.4) c.812C>T p.(Ala271Val) Heterozygous, VUS	ClinVar ID 1514580
			*COL12A1* (NM_004370.6;) seq[GRCh38] del(6)(q13q13) NC_000006.12:g.(74895844_75131521)del/1× Heterozygous, Pathogenic	[15]

Key: - = absent; C = consanguinity; F = female; M = male; MIM = Mendelian inheritance in man; NDD = neurodevelopmental disorder; SNHL = sensorineural hearing loss; VUS = variant of uncertain significance; y = year(s).

**Table 2:** Individuals with ichthyosis diagnosed in the center, including sex, age at first evaluation, clinical findings, diagnosis, and gene variant(s).

# Sex Age	C	Clinical Synopsis Diagnosis, MIM	Gene (Transcript) Variant Zygosity, Classification	Reference
7 M 7 ^0^/_12_ y	+	Collodion baby, ichthyosiform erythroderma. Autosomal recessive congenital ichthyosis 6, MIM 612281	*NIPAL4* (NM_001099287.2) c.341C>A p.(Ala114Asp) Homozygous, Pathogenic	[16]
8 M 26 ^1^/_12_ y	+	Nonbullous congenital ichthyosiform erythroderma. Autosomal recessive congenital ichthyosis 10, MIM 615024	*PNPLA1* (NM_001374623.1) c.106C>G p.(Arg36Gly) Homozygous, VUS	novel
9 F 8 ^7^/_12_ y	+	Generalized ichthyosis. Autosomal recessive congenital ichthyosis 13, MIM: 617574	*SDR9C7* (NM_148897.3) c.454C>T p.(Arg152Trp) Homozygous, VUS	[17]
10 F 3 ^1^/_12_ y	+	Pruritic ichthyosis, lower limbs spasticity, NDD. Sjögren–Larsson syndrome, MIM 270200	*ALDH3A2* (NM_000382.3) c.1443+1G>A p.? Homozygous, Likely Pathogenic	ClinVar ID 555455

Key: + = present; C = consanguinity; F = female; M = male; MIM = Mendelian inheritance in man; NDD = neurodevelopmental disorder; VUS = variant of uncertain significance; y = year(s).

**Table 3:** Individuals with albinism diagnosed in the center, including sex, age at first evaluation, clinical findings, diagnosis, and gene variant(s).

# Sex Age	C	Clinical Synopsis Diagnosis, MIM	Gene (Transcript) Variant Zygosity, Classification	Reference
11 F ^1^/_12_ y	+	Generalized skin and hair hypopigmentation, pink irides, nystagmus, photophobia. Oculocutaneous albinism type IA, MIM: 203100	*TYR* (NM_000372.5) c.872G>A p.(Gly291Glu) Homozygous, VUS	novel
12 M ^1^/_12_ y	-	Generalized skin and hair hypopigmentation, pink irides, nystagmus. Oculocutaneous albinism type IA, MIM: 203100	*TYR* (NM_000372.5) c.1036+2T>G p.(?) Heterozygous, Pathogenic	[18]
			*TYR* (NM_000372.5) c.865T>C p.(Cys289Arg) Heterozygous, Likely Pathogenic	[19,20]
13 F 2 ^11^/_12_ y	-	Generalized skin and hair hypopigmentation, brown irides, nystagmus, postaxial polydactyly in hands, mild NDD, ASD. Oculocutaneous albinism type II and neurodevelopmental disorder with seizures and nonepileptic hyperkinetic movements, MIM: 203200 and 618497	*OCA2* (NM_000275.3) c.1441G>A p.(Ala481Thr) Heterozygous, VUS	[21]
			*OCA2* (NM_000275.3) Seq[GRCh38] dup(15)(q13.1q13.1) NC_000015.10:g. (27898042_28040209)dup/3× Heterozygous, VUS	[22]
			*CACNA1B* (NM_000718.4) c.2860C>T p.(Arg954Trp) Heterozygous, VUS	novel
			*CACNA1B* (NM_000718.4) c.3275C>T p.(Thr1092Met) Heterozygous, VUS	novel
14 M 2 ^8^/_12_ y	-	Generalized skin and hair hypopigmentation, pink irides, nystagmus, severe NDD, abnormal gait, behavioral abnormalities, ASD. Oculocutaneous albinism type II and Angelman syndrome, MIM: 203200 and 105830	*OCA2* (NM_000275.3) seq[GRCh38] del(15)(q13.1q13.1) NC_000015.10:g. (28017719_28020677)del/1× Homozygous, Pathogenic	[22]
			Loss of Heterozygosity chr15: 1-101.991.189 [hg38] Pathogenic	[23]

Key: - = absent; + = present; ASD = autism spectrum disorder; C = consanguinity; F = female; M = male; MIM = Mendelian inheritance in man; NDD = neurodevelopmental disorder; VUS = variant of uncertain significance; y = year(s).

**Table 4:** Individuals with tuberous sclerosis complex diagnosed in the center, including sex, age at first evaluation, clinical findings, diagnosis, and gene variant(s).

# Sex Age	C	Clinical Synopsis Diagnosis, MIM	Gene (Transcript) Variant Zygosity, Classification	Reference
15 M 3 ^8^/_12_ y	-	Epilepsy, severe neurologic regression, subependymal nodules, facial angiofibroma, hypomelanotic macules, confetti-like lesions. Tuberous sclerosis-1, MIM 191100	*TSC1* (NM_000368.5) c.1759A>T p.(Lys587Ter) Heterozygous, Pathogenic	[24]
16 M 6 ^1^/_12_ y	-	Epilepsy, subependymal nodules, NDD, facial angiofibroma, confetti-like lesions, hypomelanotic macules, enamel pit. Tuberous sclerosis-2, MIM 613254	*TSC2* (NM_000548.5) c.1790A>G p.(His597Arg) Heterozygous, Likely Pathogenic	[25]
17 M 1 ^0^/_12_ y	-	Epilepsy, subependymal nodules, NDD, hypomelanotic macules. Tuberous sclerosis-2, MIM 613254	seq[GRCh38] del(16)(p13.3p13.3) NC_000016.10:g.(2046887_2068788)del/1× Heterozygous, Pathogenic	[26]
18 M ^3^/_12_ y	-	Cardiac rhabdomyoma, subependymal nodules, hypomelanotic macules. Tuberous sclerosis-2, MIM 613254	*TSC2* (NM_000548.5) seq[GRCh38] del(16)(p13.3p13.3) NC_000016.10:g. (2054450_2056500)del/1× Heterozygous, Pathogenic	[26]

Key: - = absent; C = consanguinity; F = female; M = male; MIM = Mendelian inheritance in man; NDD = neurodevelopmental disorder; y = year(s).

**Table 5:** Individuals with incontinentia pigmenti diagnosed in the center, including sex, age at first evaluation, clinical findings, diagnosis, and gene variant(s).

# Sex Age	C	Clinical Synopsis Diagnosis, MIM	Gene (Transcript) Variant Zygosity, Classification	Reference
19 F 2 ^0^/_12_ y	-	Neonatal vesicles progressing to verrucous lesions and hyperpigmented blaschkoid streaks, right inferior premolar agenesis. Incontinentia pigmenti, MIM 308300	*IKBKG* (NM_001099857.5) seq[GRCh38] del(X)(q28q28) NC_000023.11:g. (154557674_154569357)del/1× Heterozygous, Pathogenic	[27]
		Selective tooth agenesis 8, MIM 617073 (?)	*WNT10B* (NM_003394.4) c.1091G>T p.(Cys364Phe) Heterozygous, VUS	novel
20 F ^4^/_12_ y	-	Neonatal vesicles progressing to verrucous lesions and hyperpigmented blaschkoid streaks. Incontinentia pigmenti, MIM 308300	*IKBKG* (NM_001099857.5) seq[GRCh38] del(X)(q28q28) NC_000023.11:g.(154557889_154569575)del/1× Heterozygous, Pathogenic	[27]
21 F 21 ^2^/_12_ y	-	Neonatal vesicles progressing to verrucous lesions/hyperpigmented/hypopigmented blaschkoid streaks, dental abnormalities. Incontinentia pigmenti, MIM 308300	*IKBKG* (NM_001099857.5) seq[GRCh38] del(X)(q28q28) NC_000023.11:g. (154557674_154569357)del/1× Heterozygous, Pathogenic	[27]

Key: - = absent; + = present; C = consanguinity; F = female; MIM = Mendelian inheritance in man; VUS = variant of uncertain significance; y = year(s).

**Table 6:** Individuals with other genodermatoses diagnosed in the center, including sex, age at first evaluation, clinical findings, diagnosis, and gene variant(s).

# Sex Age	C	Clinical Synopsis Diagnosis, MIM	Gene (Transcript) Variant Zygosity, Classification	Reference
22 F ^10^/_12_ y	-	Neonatal vesicles progressing to hyperpigmented blaschkoid streaks, epilepsy, microphthalmos, severe NDD. Transient bullous of the newborn, MIM 131705	*COL7A1* (NM_000094.4) c.58_70del p. (Arg20Serfs*6) Heterozygous, Pathogenic	ClinVar ID 1047934
23 F ^8^/_12_ y	-	Generalized loose redundant skin with excessive skin folds and aged appearance. Autosomal dominant cutis laxa, MIM 123700	*ELN* (NM_000501.4) c.2132del p. (Gly711Valfs*40) Heterozygous, Likely Pathogenic	novel
24 M 5 ^0^/_12_ y	-	Short stature, microcephaly, facial telangiectasia in midface, hyperpigmented spots on trunk, learning difficulties. Bloom syndrome, MIM 210900	*BLM* (NM_000057.4) seq[GRCh38] del(15)(q26.1q26.1) NC_000015.10:g. (90805527_90841677)del/0× Homozygous, Pathogenic	novel
25 F 12 ^9^/_12_ y	-	Macrocephaly, axillary freckling, milia, odontogenic keratocysts, NSS, ASD. Basal cell nevus syndrome 1, MIM 109400	*PTCH1* (NM_000264.5) c.2350G>T p.(Glu784Ter) Heterozygous, Likely Pathogenic	novel
26 M 12 ^4^/_12_ y	-	Facial asymmetry, coloboma, focal dermal aplasia/hypoplasia, hypopigmented streaks, split hand and foot, foot monodactyly. Focal dermal hypoplasia, MIM 305600	*PORCN* (NM_203475.3) c.31C>T p.(Gln11Ter) Heterozygous, Likely Pathogenic	novel
27 F 15 ^2^/_12_ y	+	Macrocephaly, multiple lentigines, trichilemmoma, hyperpigmented spots on the glans, intestinal polyp, NDD, ASD. Cowden syndrome 1, MIM 158350	*PTEN* (NM_000314.8) c.638del p. (Pro213Leufs*8) Heterozygous, Pathogenic	ClinVar ID 2583858
28 F 23 ^4^/_12_ y	-	Hyperpigmented macules of lower lip and buccal mucosa, hamartomatous gastric and rectal polyps. Peutz–Jeghers syndrome, MIM 175200	*STK11* (NM_000455.5) c.790_793del p.(Phe264Argfs*22) Heterozygous, Pathogenic	[28]
29 F 19 ^10^/_12_ y	-	Severe short stature, cachexia, deeply set eyes, progeroid facial appearance, hyperpigmented nevi on sun-exposed areas, cerebral calcification, leukoencephalopathy, seizures, mitral valve prolapse, bilateral SNHL. Cockayne syndrome type B, MIM 133540	*ERCC6* (NM_000124.4) c.3778+947T>A p.? Hemizygous, VUS	novel
			Seq[GRCh38] del(10)(q11.22q11.23) NC_000010.11:g.(45796862_50151726)del/1× Heterozygous, Pathogenic	novel
30 M 25 ^10^/_12_ y	-	Panhypopituitarism, facial telangiectases, pulmonary arteriovenous malformation, lower limbs ochre dermatitis. Telangiectasia hereditary hemorrhagic, MIM 187300	*ENG* (NM_001114753.3) c.1832G>A p.(Trp611Ter) Heterozygous, VUS	novel
31 M 2 ^10^/_12_ y	-	Short stature, micrognathia, short nose, premature loss of teeth, cataract, poikiloderma, frontal balding, cutis marmorata, mild NDD. Rothmund–Thomson syndrome?	Negative test	
32 M 1 ^9^/_12_ y	-	Mild NDD, asymmetric face, limb hypertrophy, macrodactyly, syndactyly, diffuse cutaneous hemangiomata, body asymmetry. Klippel–Trénaunay syndrome, MIM 149000	Negative test	
33 F 9 ^0^/_12_ y	-	Borderline NDD, epilepsy, intracranial calcifications, trigeminal hemangiomata, trunk and limb hemangiomata, body asymmetry. Sturge–Weber + Klippel–Trénaunay syndrome, MIM 185300 + 149000	Negative test	

Key: - = absent; + = present; ASD = autism spectrum disorder; C = consanguinity; F = female; M = male; MIM = Mendelian inheritance in man; NDD = neurodevelopmental disorder; SNHL = sensorineural hearing loss; VUS = variant of uncertain significance; y = year(s).

## Data Availability

The data supporting the findings of this study are available from the corresponding author upon reasonable request.

## Abbreviations

The following abbreviations are used in this manuscript:

ASD	Autism spectrum disorder
BRGP	Brazilian Rare Genomes Project
C	Consanguinity
CGS/Unicamp	Clinical Genetics Service at the University of Campinas
FROH	Fraction of runs of homozygosity
NDD	Neurodevelopmental disorder
MIM	Mendelian inheritance in man
NROH	Number of runs of homozygosity
RASopathies	Disorders comprising genes that encode components or regulators of the Ras/mitogen-activated protein kinase (MAPK) pathway
SNHL	Sensorineural hearing loss
SROH	Sum of runs of homozygosity
SUS	Sistema Único de Saúde (Unified Health System)
VUS	Variant of uncertain significance
